# Comparison of the efficacy of LTCBDE and LCBDE for common bile duct stones: a systematic review and meta-analysis

**DOI:** 10.3389/fsurg.2024.1412334

**Published:** 2025-01-08

**Authors:** Bin Zheng, Yixin Lu, Erqi Li, Ziyu Bai, Kaiqian Zhang, Jian Li

**Affiliations:** ^1^ Department of Hepatobiliary Surgery, Affiliated Hospital of Chengde Medical University, Chengde, Hebei Province, China; ^2^ Department of Cardiovascular Medicine, Affiliated Hospital of Chengde Medical University, Chengde, Hebei Province, China; ^3^ Hebei Key Laboratory of Panvascular Diseases, Chengde, China

**Keywords:** surgery, LTCBDE, LCBDE, CBDS, meta-analysis

## Abstract

**Background:**

The choice of surgical methods for common bile duct stones (CBDS) is controversial. The aim of this study was to compare the safety and efficacy of laparoscopic transcystic common bile duct exploration (LTCBDE) and laparoscopic common bile duct exploration (LCBDE).

**Methods:**

Relevant literature published before March 30, 2023 in PubMed, Web of Science, Embase, and Cochrane was searched to screen studies comparing LTCBDE and LCBDE. RevMan 5.4 was used for meta-analysis of fixed-effects and random-effects models.

**Results:**

A total of 21 studies met the inclusion criteria, including 3065 patients in the LTCBDE group and 2,453 patients in the LCBDE group. CBDS clearance was 95.4% (2,682/2,812) in LTCBDE group and 94.7% (1,810/1,911) in LCBDE group (OR: 1.84, 95% CI: 1.36, 2.48, *P* < 0.0001; *I*^2^ = 0%, *P* = 0.56). In LTCBDE group, operative time(MD = −34.60, 95% CI: −46.05, −23, 15, *P* < 0.00001 *I*^2^ = 96%, *P* < 0.00001), postoperative hospital stay (MD = −2.92, 95% CI: −3.62, −2.21, *P* < 0.00001; *I*^2^ = 92%, *P* < 0.00001), postoperative complications (OR: 0.47, 95% CI: 0.38, 0.58, *P* < 0.0001; *I*^2^ = 26%, *P* = 0.15), residual stone(OR: 0.48, 95% CI: 0.34, 0.66, *P* < 0.0001; *I*^2^ = 0%, *P* = 0.56), bile leak (OR: 0.37, 95% CI: 0.25, 0.55, *P* < 0.00001; *I*^2^ = 0%,*P* = 0.52), mortality (OR: 0.10, 95% CI: 0.01, 0.88, *P* = 0.04; *I*^2^ = 0%, *P* = 0.71) and recurrent stones(OR: 0.34, 95% CI: 0.15, 0.74, *P* = 0.007; *I*^2^ = 5%, *P* = 0.38) were better than LCBDE group. There was no difference in pancreatitis (OR: 1.06, 95% CI: 0.52, 2.16. *P* = 0.86; *I*^2^ = 0%, *P* = 0.98) and biliary stricture(OR: 0.30, 95% CI: 0.08, 1.09, *P* = 0.07; *I*^2^ = 0%, *P* = 0.57).

**Conclusions:**

LTCBDE is safe, efficient, and of great clinical significance, and is worth promoting to some patients.

## Introduction

CBDS are one of the most common biliary tract diseases, accounting for approximately 10%–15% of the total number of patients with cholelithiasis (1). CBDS can cause adverse complications such as obstructive jaundice, cholangitis, liver abscess, pancreatitis and secondary biliary cirrhosis, which have a serious impact on the physical and mental health of patients.

At present, the main ways we take to treat CBDS are: laparoscopic or open choledochotomy and exploration, endoscopic retrograde cholangiopancreatography (ERCP) + internal sphincterotomy and stone extraction. Endoscopic sphincterotomy is prone to cause loss of sphincter of Oddi function, so some scholars believe that the disadvantages of this procedure outweigh the advantages (2). T-tube was firstly used in biliary surgery in 1889, because it has the advantages of smooth biliary drainage, supporting the biliary tract to avoid stenosis, and secondary extraction of stones through the T-tube sinus tract and so on, so it has been used until now. However, the drainage of T-tube will cause a large amount of bile salts, electrolytes, water loss, which is not in line with the normal physiological state of the human body, and a large number of experiments have confirmed that placing the T-tube is easy to affect the duodenal bacteria, food reflux choledochotomy and cause secondary infections (3). Scholars at home and abroad have different attitudes towards whether to leave a T-tube or not. 1897 Halsted advocated the primary duct closure (PDC) of the biliary tract, but due to the objective limitations at that time, it was difficult to become the mainstream knowledge. Some scholars have shown that one-stage suture can reduce postoperative complications better than indwelling T-tube, and it should be the preferred procedure for the treatment of bile duct stones (4). With the gradual improvement of medical technology and hardware facilities at home and abroad, the precise evaluation of preoperative MRCP has a high reference value for the choice of surgical strategy for CBDS (5), which makes the application of LCBDE + PDC more widely.

With the maturation of the laparoscopy technique, LTCBDE was first reported in detail by scholars in the United States in 1992, and the technique has been gradually and widely performed abroad. LTCBDE is undoubtedly the least invasive, safest, and most effective option for the treatment of CBDS (6). LTCBDE avoids choledochotomy and eliminates the subsequent requirement for a T-tube, and the transcystic approach is less damaging than laparoscopic choledochotomy and maintains the integrity of the common bile duct (CBD). For patients with gallbladder stones (GS) and CBDS, LTCBDE has the advantages of less trauma, good stone extraction, fewer complications, and faster postoperative recovery (7). However, LTCBDE has not been generally accepted, and there are not enough large multicenter studies to investigate its safety and efficacy. There are also no clear guidelines on the indications for LTCBDE and LCBDE. The purpose of this study was to perform a pooled analysis of published data on LTCBDE and LCBDE with the aim of analyzing and comparing the efficacy, complications, and feasibility of the two procedures.

## Materials and methods

This study is fully compliant with the Preferred Reporting Items for Systematic Reviews and Meta-analyses (PRISMA) statement and registered with PROSPERO (CRD42023439202). The Ethics Committee of the Affiliated Hospital of Chengde Medical University approved the project and obtained written form of consent (CYFYLL2022476).

Relevant literature published before March 30, 2023 in PubMed, Web of Science, Embase, and Cochrane was searched to screen studies comparing LTCBDE and LCBDE without language restrictions. The specific search strategy was as follows:“([LTCBDE(Title/Abstract)] OR [Laparoscopic transcystic common bile duct exploration(Title/Abstract)] OR [Laparoscopic common bile duct exploration(Title/Abstract)]) OR [LCBDE(Title/Abstract)]) AND ([Cholelithiasis, Common Bile Duct(Title/Abstract )] OR [“Choledocholithiasis” (Mesh)]).” In addition, a manual search of the references of all retrieved reviews was performed to find additional studies for possible inclusion.

### Inclusion and exclusion criteria

#### Inclusion criteria

(1) No restrictions on randomized controlled trials, retrospective cohort studies, or prospective cohort studies; (2) Clinical studies comparing LTCBDE with LCBDE; (3) Clinical diagnosis consistent with GS combined with CBDS, and imaging evidence of GS combined with CBDS; (4) Studies including at least one outcome metric (CBDS clearance rate, total complications, bile leak, stone retention, stone recurrence, bile duct stricture, operative time, and postoperative hospital stay); (5) For more than one literature from the same institution, the most recent or complete literature was selected for meta-analysis.

#### Exclusion criteria

(1) History of cholecystectomy; (2) Uncontrolled and animal studies; (3) Abstracts, case reports, letters, or reviews only; (4) Articles that reused data or did not have sufficient data. (5) Articles in languages other than English.

### Data extraction and outcomes of interest

Data were independently extracted and checked by 2 evaluators and data discrepancies were resolved by reference to relevant knowledge and discussion. Baseline characteristics of study patients, surgical data, and postoperative outcomes were used to compare LTCBDE with LCBDE. Baseline characteristics of study patients included first author, year of publication, country, study type, number of patients in each group, age, and gender. Surgical outcomes included CBDS clearance, operative time, intraoperative bleeding, and operative technique. Postoperative outcome observables included postoperative hospitalization time, total complications, bile leakage, pancreatitis, stone retention, stone recurrence, and biliary stricture. CBDS removal, bile leak, and total complications were our primary outcomes. Total complications were defined as including residual stone, bile leak, pancreatitis, death, stone recurrence, bile duct stricture, hyperamylasemia, postoperative hemorrhage, incision infection, pneumonia, and T-tube related complications.

### Quality assessment

The search results were evaluated independently by 2 authors, and RCT studies were assessed for risk of bias using the Cochrane Risk of Bias Assessment Tool (RoB2). Retrospective and prospective cohort studies were assessed for quality using the modified Newcastle-Ottawa Scale (NOS), and each cohort study was rated on a scale of 0–9, indicated by an asterisk, and studies with at least 6 asterisks were considered high-quality studies.

### Statistical analysis

Meta-analysis was performed using RevMan 5.4 (Cochrane collaboration, Oxford, England). Cochran's Q test and Higgins *I*^2^ statistic were used to evaluate inter-study heterogeneity. When no significant heterogeneity was detected (*I*^2^ ≤ 50% or *P* ≥ 0.10), we used a fixed-effects model. When significant heterogeneity was present (*P* < 0.10 or *I*^2^ > 50%), a random effects model was used. Dichotomous variables were analyzed and assessed using the ratio of ratios (OR), and continuous variables were analyzed using weighted mean differences. Differences were considered statistically significant at *P* < 0.05. Ninety-five percent confidence intervals (CI) are reported for all results. When continuous variables were reported as median and range or median and IQR, the mean and standard deviation (SD) were estimated using the method described by Luo et al. (8) for meta-analysis data synthesis.

### Publication bias

This study used a funnel plot to qualitatively analyze the risk of publication bias.

## Results

### Study selection

**Figure 1:**
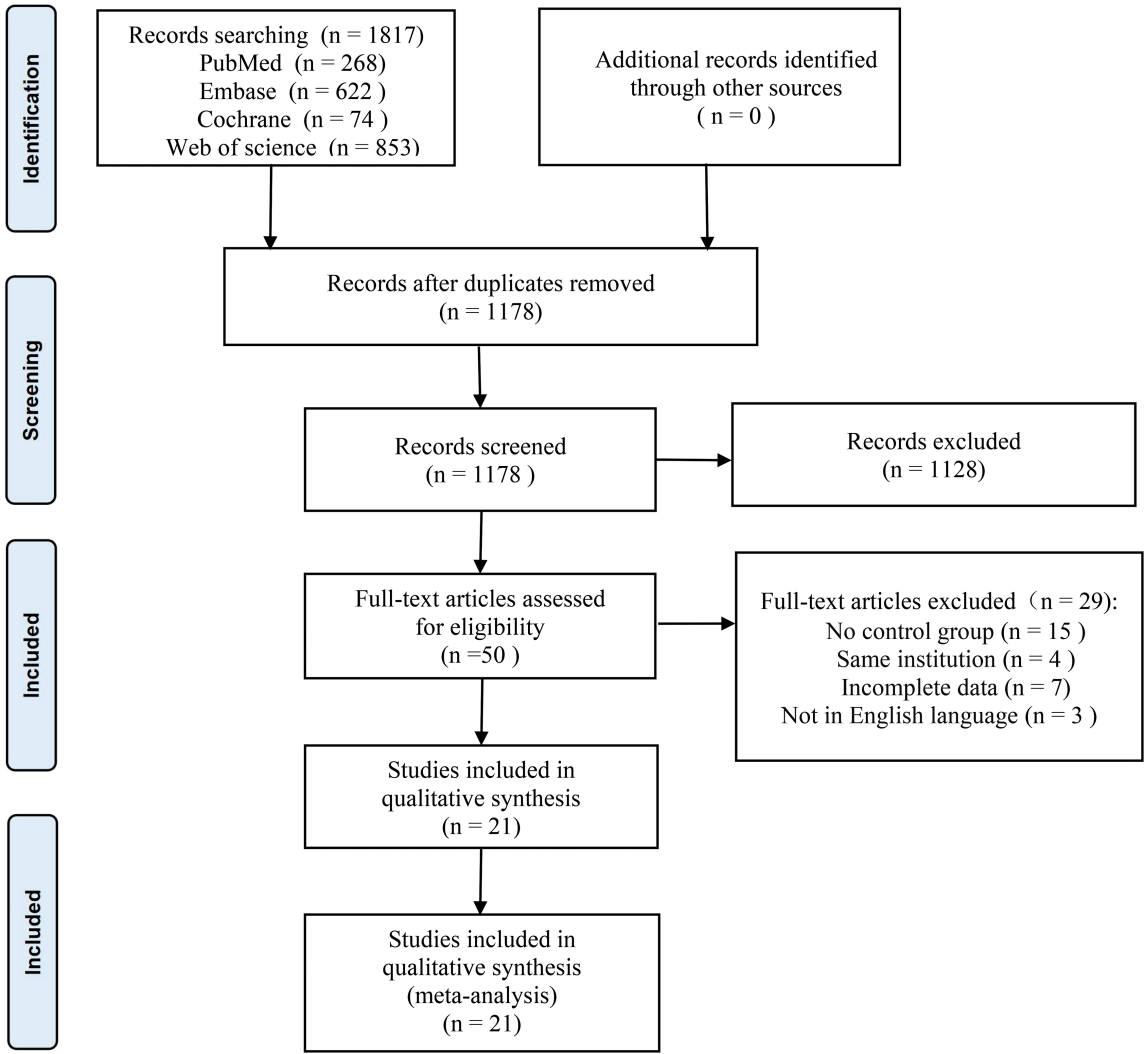
The flowchart for the study search and screening.

A total of 1817 documents were retrieved for this study, including 268 from PubMed, 622 from Embase, 74 from Cochrane, 853 from Web Of Science, and no other eligible studies were found from other sources. Duplicates were removed from 639 papers, 1,128 papers were excluded on the basis of title and abstract, the remaining 50 studies were evaluated by reading the full text, and 29 papers were further excluded (including 15 studies without a control group, 4 articles from the same institutional data, 3 papers with incomplete data, and 3 non-English studies). Twenty-one papers were finally included (Martin 1998 (9), Rhodes 1998 (10), Cuschieri 1999 (11), Lauter 2000 (12), Waage 2003 (13), Paganini 2007 (14), Topal 2007 (15), ElGeidie 2011 (16), Grubnik 2012 (17), Chen 2013 (18), Poh 2014 (19), Huang 2015 (20), Zhang 2015 (21), Aawsaj 2016 (22), Mattila 2017 (23), Quaresima 2017 (24), Al- Temimi 2019 (25), Al-Ardah 2021 (26), Guo 2022 (27), Nassar 2022 (28), Zhu 2022 (29). Among them, 4 RCTs Rhodes 1998, Cuschieri 1999, ElGeidie 2011, Grubnik 2012, and the remaining 17 were non-RCTs), including 3,065 patients with LTCBDE and 2,453 patients with LCBDE. Figure 1 illustrates the PRISMA flowchart of the literature search strategy. Table 1 shows the baseline characteristics of the 21 included studies. Supplementary Table S1 shows the clinical outcomes of the included studies. Supplementary Table S2 shows the complications of the included studies.

**Table 1:** Baseline characteristics of the 21 studies included.

Study	Year	Country	Desige	LTCBDE (Number)	LCBDE (Number)	LTCBDE age (years)	LCBDE age (years)	LTCBDE Gender (M/F)	LTCBDE Gender(M/F)
					TTD/PDC		TTD/PDC		(TTD)/(PDC)
Martin et al. (8)	1991−1997	AUS	Non-RCT	158	61/55	48 (19–100)	56 (19–94)/52 (24–83)	–	–
Rhodes et al. (10)	1995–1997	GBR	RCT	28	12	–	–	–	–
Cuschieri et al. (11)	1994–1997	GBR	RCT	56	53	–	–	–	–
Lauter and Froines (12)	1994–1999	USA	Non-RCT	26	0/25	–	–	–	–
Waage et al. (13)	1992–1999	SWE	Non-RCT	110	52/0	–	–	–	–
Paganini et al. (14)	1991–2004	ITA	Non-RCT	191	138	54.5 (12–88）	–	67/124	–
Topal et al. (15)	2001–2006	BEL	Non-RCT	83	30	62 (15–86)	76 (18–86)	28/55	11/19
ElGeidie et al. (16)	2009–2010	EGY	RCT	57	49	–	–	–	–
Grubnik et al. (17)	2005–2009	UKR	RCT	76	62	56.5	66/-	–	–
Chen et al. (18)	2009–2013	CHN	Non-RCT	110	100	48 ± 11	50 ± 12.9	20/90	30/70
Poh et al. (19)	2010–2012	AUS	Non-RCT	80	3	–	–	–	–
Huang et al. (20)	2008–2012	CHN	Non-RCT	80	0/209	60 ± 16.7	−/58 ± 16.63	52/28	−/(111/98)
Zhang et al. (21)	2000–2009	CHN	Non-RCT	237	46/47	54.7 ± 13.3	52.0 ± 15.9/52.3 ± 16.6	98/139	(19/27)/(22/25)
Aawsaj et al. (22)	2000–2015	GBR	Non-RCT	63	233	–	–	–	–
Mattila et al. (23)	1999–2014	FIN	Non-RCT	64	33	–	–	–	–
Quaresima et al. (24)	1991–2014	ITA	Non-RCT	214	170	57 (24–96)	67 (12–88)	79/135	72/98
Al-Temimi et al. (25)	2005–2015	USA	Non-RCT	103	12	50.6 ± 21.9	74.0 ± 15.6	30/73	0/12
Al-Ardah et al. (26)	2006–2019	GBR	Non-RCT	111	68	43.8 ± 16.7	47.1 ± 17.1	21/90	16/52
Guo et al. (27)	2013–2019	CHN	Non-RCT	280	0/479	52.1 ± 15.06	−51.65 ± 16.07	138/142	−/(268/211)
Nassar et al. (28)	1992–2020	GBR	Non-RCT	870	448	–	–	–	–
Zhu et al. (29)	2007–2018	CHN	Non-RCT	68	68	64 (52–73)	66 (58–72)	30/38	31/37

Year, indicates the start and end time of the study; Age, is expressed as mean ± standard deviation (SD) or median (range); LTCBDE, laparoscopic transcystic common bile duct exploration; LCBDE, laparoscopic common bile duct exploration; PDC, primary duct closure; TTD, T-tube drainage; M, male; F, female; RCT, randomized controlled trial; Non-RCT, non randomized controlled trial; CHN, China; AUS, Australia; GBR, United Kingdom of Great Britain and Northern Ireland; USA, The United States of America; SWE, Sweden; ITA, Italy; BEL, Belgium; EGY, Egypt; UKR, Ukraine; FIN, Finland.

### Study characteristics and quality assessment

A comparison of the LTCBDE and LCBDE technical processes across studies is shown in Supplementary Table S3. RCT studies were assessed for risk of bias using the Cochrane Risk of Bias Assessment Tool (RoB2) (Supplementary Figure S1). Retrospective and prospective cohort studies were assessed for quality using the modified Newcastle-Ottawa Scale (NOS) (Supplementary Table S4).

#### CBDS clearance

**Figure 2:**
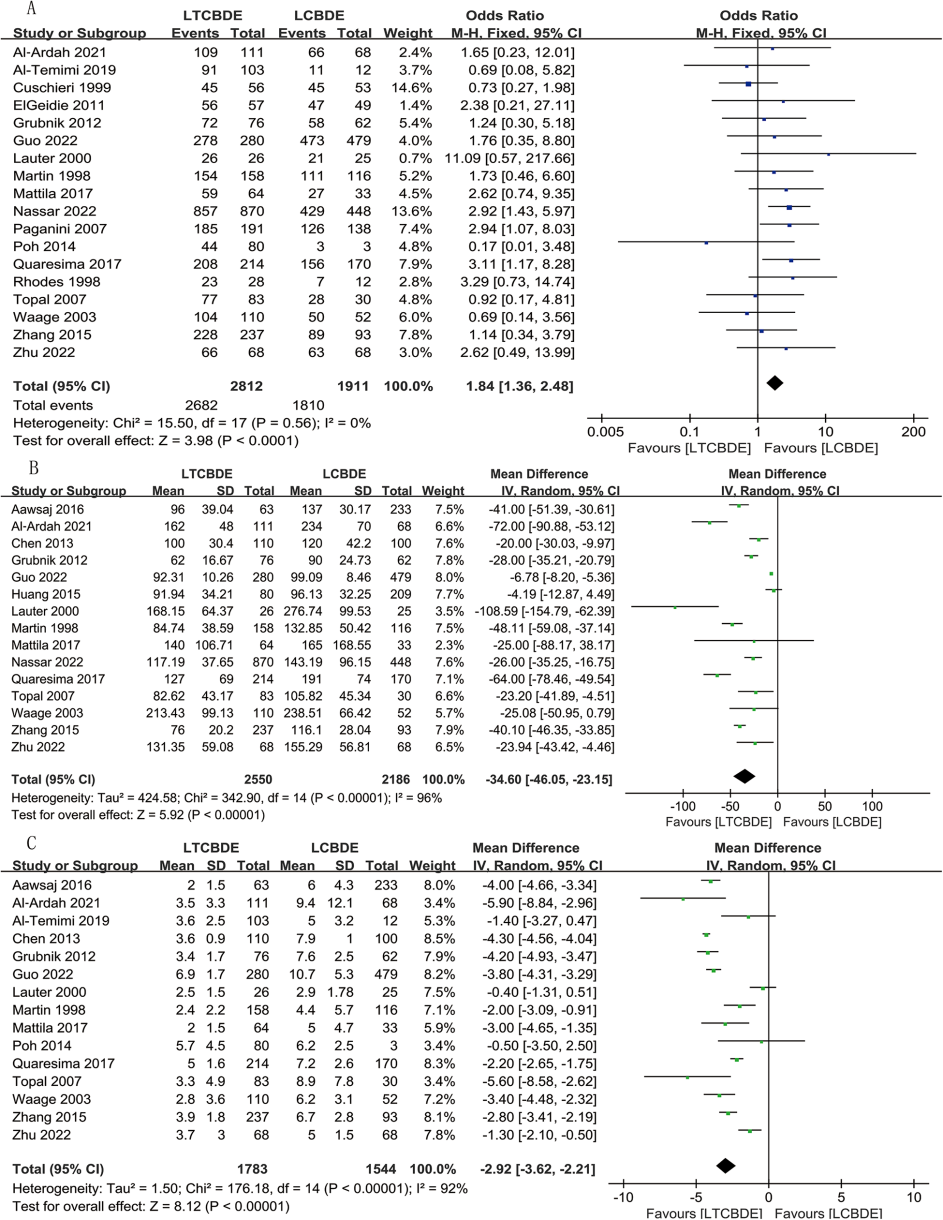
Forest plot of surgical related data study results. **(A)** CBDS clearance; **(B)** Operative time; **(C)** Postoperative hospital stay.

18 papers reported this result, and analysis using a fixed model showed that complete clearance of CBDS was observed in 95.4% of patients in the LTCBDE group (2,682/2,812) and in 94.7% of patients in the LCBDE group (1,810/1,911). The difference between the two groups was statistically significant (OR: 1.84, 95% CI: 1.36, 2.48, *P* < 0.0001; *I*^2^ = 0%, *P* = 0.56) (Figure 2A).

#### Operative time

A total of 15 studies were included, and the application of random-effects model analysis showed that the operative time (min) was shorter in LTCBDE compared with LCBDE, and the difference between the two groups was statistically significant (MD = −34.60, 95% CI: −46.05, −23, 15, *P* < 0.00001; *I*^2^ = 96%, *P* < 0.00001) (Figure 2B).

#### Postoperative hospital stay

15 studies reported the results, and a combined analysis using a random-effects model showed that the postoperative hospital stay(d) was shorter with the application of LTCBDE than with the application of LCBDE, and the difference between the two groups was statistically significant (MD = −2.92, 95% CI: −3.62, −2.21, *P* < 0.00001; *I*^2^ = 92%, *P* < 0.00001) (Figure 2C).

#### Postoperative complications

**Figure 3:**
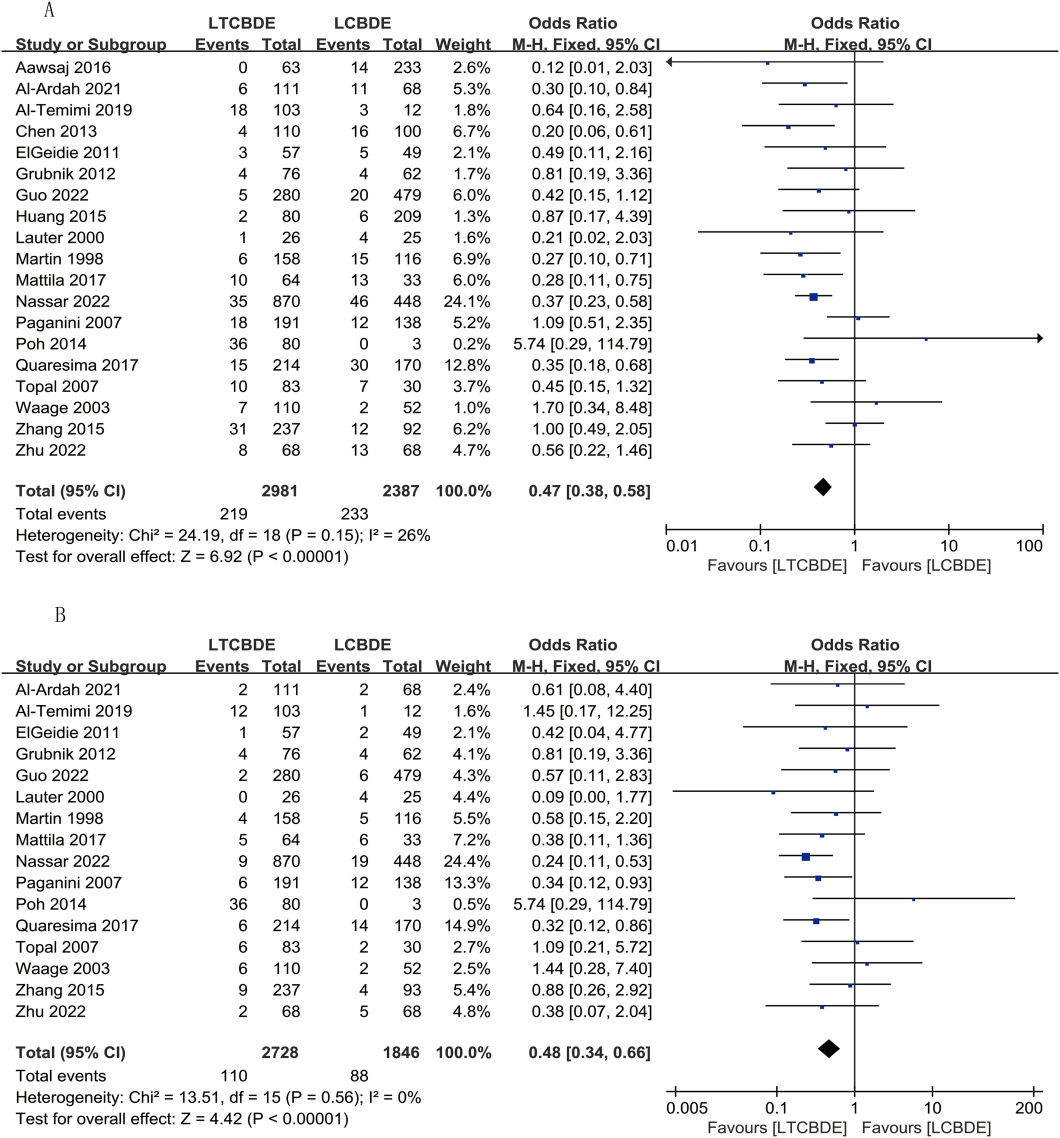
Forest plot of postoperative complication study results. **(A)** Postoperative complications; **(B)** Residual stone.

A total of 19 papers were included in the results of this study, and the application of fixed-effects model analysis showed that the total postoperative complication rate was significantly lower in the LTCBDE group than in the LCBDE group, and the difference between the two groups was statistically significant (OR: 0.47, 95% CI: 0.38, 0.58, *P* < 0.0001; *I*^2^ = 26%, *P* = 0.15) (Figure 3A).

#### Residual stone

16 studies reported this result, and the application of fixed-effects model analysis showed that the incidence of residual stones was significantly lower in the LTCBDE group than in the LCBDE group, and the difference between the two groups was statistically significant (OR: 0.48, 95% CI: 0.34, 0.66, *P* < 0.0001; *I*^2^ = 0%, *P* = 0.56) (Figure 3B).

#### Bile leak

**Figure 4:**
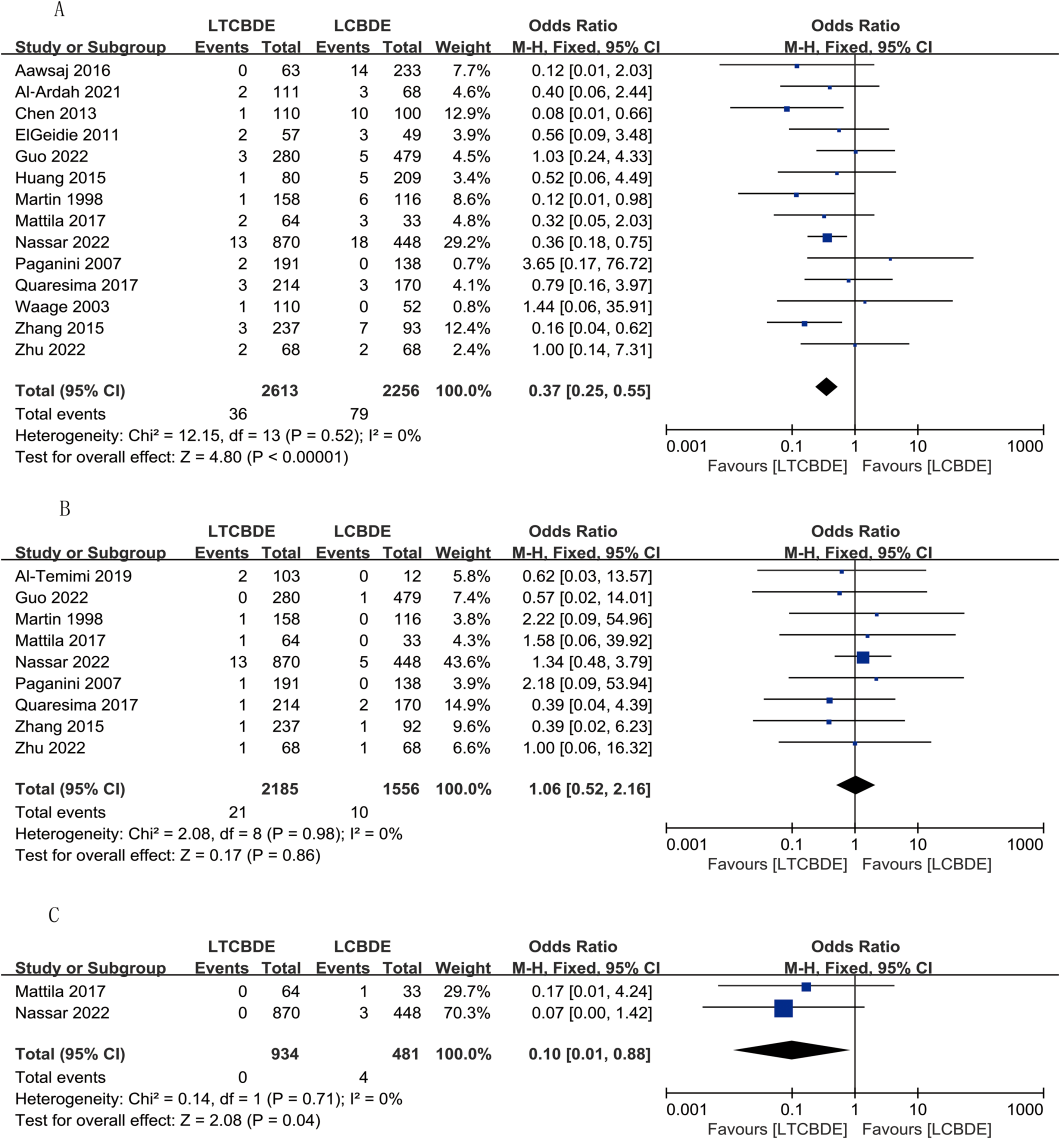
Forest plot of postoperative complication study results. **(A)** Bile leak; **(B)** Pancreatitis; **(C)** Mortality.

A total of 14 studies were included, and analysis of the results applying a fixed-effects model showed a lower incidence of postoperative bile leakage in LTCBDE compared with LCBDE. The difference between the two groups was statistically significant (OR: 0.37, 95% CI: 0.25, 0.55, *P* < 0.00001; *I*^2^ = 0%, *P* = 0.52) (Figure 4A).

#### Pancreatitis

A total of 9 papers were included, and analysis of the results applying a fixed-effects model showed that the incidence of postoperative pancreatitis was higher in the LTCBDE group than in the LCBDE group, but the difference between the two groups was not statistically significant (OR: 1.06, 95% CI: 0.52, 2.16, *P* = 0.86; *I*^2^ = 0%, *P* = 0.98) (Figure 4B).

#### Mortality

The results of this study, which included only 2 papers, were analyzed by applying the fixed-effects model, which showed that the perioperative mortality rate was lower in the LTCBDE group than in the LCBDE group, and the difference between the two groups was statistically significant (OR: 0.10, 95% CI: 0.01, 0.88, *P* = 0.04; *I*^2^ = 0%, *P* = 0.71) (Figure 4C).

#### Recurrent stones

**Figure 5:**
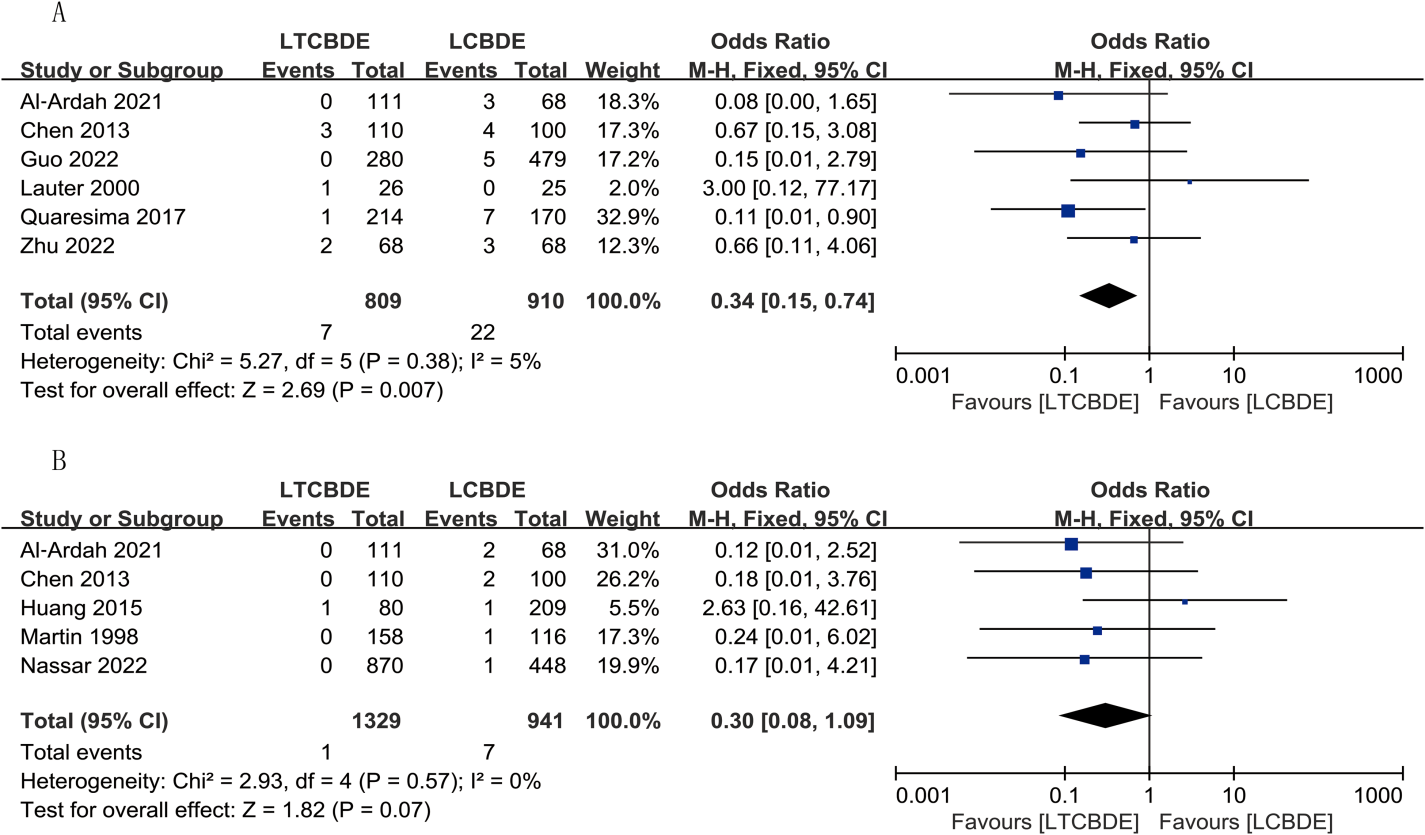
Forest plot of postoperative complication study results. **(A)** Recurrent stones; **(B)** Biliary stricture.

6 studies reported this result, and the application of fixed-effects model analysis showed that the incidence of recurrent stones was significantly lower in the LTCBDE group than in the LCBDE group, and the difference between the two groups was statistically significant (OR: 0.34, 95% CI: 0.15, 0.74, *P* = 0.007; *I*^2^ = 5%, *P* = 0.38) (Figure 5A).

#### Biliary stricture

A total of 5 papers were included in this result, and the application of fixed-effects model analysis showed that the incidence of biliary stricture was lower in the LTCBDE group than in the LCBDE group when comparing the two groups, but the difference between the two groups was not statistically significant (OR: 0.30, 95% CI: 0.08, 1.09, *P* = 0.07; *I*^2^ = 0%, *P* = 0.57) (Figure 5B).

### Publication bias

We used funnel plots to analyze the risk of bias in assessing the outcomes of CBDS clearance, operative time, postoperative hospital stay, postoperative complications, residual stone, and bile leak. Our analysis showed that the funnel plot display for all outcomes had a relatively symmetrical plot. Therefore, we concluded that there was no significant publication bias in this study (Supplementary Figure S2).

## Discussion

GS combined with CBDS is one of the common diseases in hepatobiliary surgery, there are three standard surgical treatments available, and none has been unequivocally proven superior. Currently, the main methods for the treatment of choledochal stones are LCBDE + Laparoscopic cholecystectomy (LC) and preERCP + LC (17, 30). ERCP cuts the sphincter of Oddi by endoscopic manipulation, and then applies a mesh basket to remove the CBDS. This results in the destruction of the physiological structure of the sphincter of Oddi, which may cause reflux of digestive juices leading to cholangitis, CBDS (2, 31). Moreover, ERCP has a higher incidence of other postoperative complications, including pancreatitis and biliary tract infection, and a lower stone clearance rate (6, 32). In addition, ERCP requires a two-stage procedure. The results of one study showed that nearly half of the patients with recurrent CBDS experienced stone recurrence after ERCP during long-term follow-up (33).

In recent years, an increasing number of researchers have advocated the preferred use of LCBDE as a treatment for GS combined with CBDS (22). LCBDE has more significant advantages over ERCP, offering greater cost-effectiveness as well as lower hospitalization time (34). However, there are potential risks associated with LCBDE, with T-tube drainage leading to (1) Prolonged patient hospitalization, (2)Fluid imbalance due to bile loss, (3) Increased rates of biliary tract infections, and (4) Consequential complications due to T-tube migration (3, 21, 35). A large body of evidence has demonstrated the advantages of LTCBDE over LCBDE (36), which is performed by making an incision in the cystic duct and placing a choledochoscope and a mesh basket for stone extraction through this incision, rather than directly incising through the wall of the common bile duct, which would greatly reduce complications such as bile duct stenosis and bile leakage. However, due to the anatomical characteristics of the CBD and the limitation of the characteristics of the stones, the success rate of LTCBDE + LC is often between 55% and 85% (37, 38). According to the results of related studies (27), LC + LTCBDE is often applied to patients with a diameter of the cystic duct >3 mm, the number of stones in the common bile duct <5, or the diameter of the stones <2 cm, which is a more demanding condition for patients. Therefore, the aim of this study was to perform a pooled analysis of published data on LTCBDE and LCBDE and to update the conclusions of the published meta-analysis.

The results of the meta-analysis showed that 95.4% of the patients (2,682/2,812) in the LTCBDE group and 94.7% of the patients (1,810/1,911) in the LCBDE group had their CBDS completely removed, and the difference between the two groups was statistically significant (*P* < 0.0001), and the heterogeneity between the groups was very low, which indicated that the results were reliable. The success rate of stone removal in both groups in this meta-analysis was higher than that in the meta-analysis reported by Feng et al. 2016 (39), and the success rate of stone removal was similar in both groups when compared with the meta-analysis reported by Pang et al. 2018 (40). However, the results of the present study differed from the results of the above 2 meta-analysis studies in that the results of the present study showed a higher stone extraction success rate in LTCBDE than in LCBDE, and the difference between the two groups was statistically significant (*P* < 0.0001). Our findings showed a failure rate of 4.6% in the LTCBDE group, As reported in a related study (27), unlike the traditional method of dealing with the incision at the cystic duct, the cystic duct was transected 1–2 cm from the common bile duct, and the cystic duct was dilated with a catheter or balloon. If the diameter of the cystic duct was <5 mm, a 3 mm choledochoscope was placed through the incision to explore the common bile duct. If the diameter of the cystic duct was ≥5 mm, a 5 mm choledochoscope was used. In patients with stone diameter/choledochal duct diameter ≥1, a T-shaped incision was made at the junction of the choledochal duct and CBD, and liquid electrode lithotripsy or biopsy forceps were used to fragment the stone. Stones are then routinely extracted using a mesh basket for stone extraction and saline irrigation. We believe that the increase in the success rate of stone extraction is more related to the rapid development of laparoscopic technology in recent years, the upgrade of the corresponding supporting medical equipment and the improvement of the surgeon's technical level.

For the results of surgical data, in this study, the operative time(min) was shorter in the LTCBDE group than in the LCBDE group, and the difference between the two groups was statistically significant (*P* < 0.00001). The postoperative hospitalization time(d) was shorter in the LTCBDE group than in the LCBDE group, and the difference between the two groups was statistically significant (*P* < 0.00001). The findings of the operative time and postoperative hospitalization time of this meta-analysis were consistent with those reported by 2018 Pang et al. (40) The first step of both LTCBDE and LCBDE was to expose the hepatic hilum and to dissect the gallbladder triangle, which made it possible to adequately expose the structural relationship between the cystic duct, the common hepatic duct, and the common bile duct. The LCBDE procedure then opts to make a longitudinal incision of approximately 1.0 cm in the anterior wall of the common bile duct using either micro-scissors or an electrocoagulation hook. This incision is then used to place a choledochoscope into the CBD and perform the corresponding choledochal lithotripsy operation, and then finally the gallbladder is removed by applying absorbable clips or hemo-lock to clamp the gallbladder artery and the CBD. LCBDE is divided into T-tube drainage (TTD) (13) and PDC according to the final management of the choledochotomy incision (12, 20, 27). The TTD is putting the choledochotomy with absorbable monofilament interrupted sutures and using an *in vivo* knotting technique to suture the T-tube around the choledochotomy incision, whereas PDC is a direct suture to close the choledochotomy incision directly utilizing the 3-0 Vicryl suture (all the studied technical methods can be seen in the Supplementary Table S3) The absence of choledochotomy in the LTCBDE allows for a more streamlined approach to the choledochotomy, which may shorten some of the operative time accordingly. The heterogeneity *I*^2^ for both operative time and postoperative hospitalization time in this study was greater than 50%, with a significant difference at *P* < 0.10. We performed a regression analysis for both metrics and unfortunately did not identify the source of heterogeneity. Sensitivity analyses (Supplementary Figure S3) were also done separately and showed stable results for the indicators, so we consider the results to be relatively reliable.

By analyzing the data of this study, the total postoperative complication rate in the LTCBDE group was significantly lower than that in the LCBDE group, and the difference between the two groups was statistically significant(*P* < 0.0001), with low heterogeneity and reliable results. We found that the results of this study were consistent with the findings of Pang et al. 2018 (40) that LTCBDE can significantly reduce the probability of postoperative complications and shorten the length of hospitalization of patients, reduce the mental and economic pressure of patients, and save medical resources. This study differs from previous studies in that this study compared the differences in individual postoperative complications between LTCBDE and LCBDE separately. The major short-term postoperative complications mainly included residual stones, biliary fistula, pancreatitis and mortality, while long-term complications included bile duct stenosis and stone recurrence. Heterogeneity between study groups for postoperative complications was low regardless of whether RCTs or cohort studies were included, suggesting that our results are reliable. We saw that the incidence of postoperative stone remnants was significantly lower in the LTCBDE group than in the LCBDE group, and the difference between the data of the two groups was statistically significant(*P* < 0.0001). This may be related to the differences in the centers’ grasp of the indications for this procedure, for example, studies from some centers concluded (27) that LTCBDE is often indicated for patients with a choledochal duct diameter of >3 mm, a number of choledochal stones <5, or a stone diameter of <2 cm. For PDC, patients with a common bile duct diameter >8 mm and no intrahepatic bile duct stones are required for the procedure.There is no relevant qualification for TTD. In contrast, Quaresima et al. 2017 (24) concluded that bile duct stones need to be <5 mm in size and <choledochal duct diameter in order for LTCBDE to be used. by reviewing Martin 1998 (9), Rhodes 1998 (10), Waage 2003 (13), Paganini 2007 (14), ElGeidie 2011 (16), Grubnik 2012 (17), Chen 2013 (18), Aawsaj 2016 (22), Mattila 2017 (23), Quaresima 2017 (24), and Guo 2022 (27) studies were analyzed, and we concluded that the indications for LTCBDE are CBD diameter < 8–10 mm, cystic duct diameter >3 mm, CBDS size <8–10 mm, and number of CBDS < 5, whereas the indications for LCBDE are CBD diameter ≥10 mm, CBDS size ≥10 mm, and number of CBDS ≥ 5. It is clear from this that LTCBDE has a significant advantage for patients with undilated CBD, small CBDS, and a small number of stones. This in turn makes it easier to remove stones from the common bile duct without residue.

Bile leak is one of the common complications after biliary surgery. A severe bile leak may be life-threatening if not detected and treated in time (41). In our study, we found that the incidence of bile leak in the LTCBDE group was lower than that of LCBDE, and the difference between the two groups was statistically significant (*P* < 0.00001). This may be due to the fact that LCBDE cuts the common bile duct and causes an artificial disruption of the physiology of the CBD. It was found that PDC bile leak were reduced compared to TTD. This may be due to the fact that continuous suturing of the bile duct strengthens the wall of the bile duct and prevents bile leak, whereas after suturing the T-tube for drainage there may be a gap, and displacement of the T-tube predisposes to bile leak (42). In addition, immature sinus tract formation due to incorrect timing of extubation may lead to bile leak and biliary peritonitis (43). LTCBDE did not incise the anterior wall of the CBD, and did not disrupt the physiological structure of the CBD, so the risk of biliary fistula is much lower than that of LCBDE, and it is safer than LCBDE, which is a reason for the effective reduction of the patient's hospitalization time. It has been reported that (44), primary close reduces postoperative bile leak in LCBDE. In addition, it is a safe and effective option for the treatment of CBDS.

Our meta-analysis showed that the difference in the incidence of postoperative pancreatitis (*P* = 0.86) and bile duct stenosis (*P* = 0.07) was not statistically significant between the LTCBDE group and the LCBDE group. LTCBDE did not increase the risk of postoperative pancreatitis in patients. However, it is worth noting that the included studies did not describe the diagnostic criteria and severity of pancreatitis in detail. The severity of postoperative pancreatitis ranges from mild to life-threatening (45). More detailed and higher quality studies on postoperative pancreatitis in LTCBDE and LCBDE are needed in the future. Although the difference in postoperative bile duct stenosis between the two groups was not statistically significant, the results favored a lower incidence of postoperative bile duct stenosis in the LTCBDE group. It has been suggested that more rapid necrotic evacuation and removal of inflammatory focus may reduce local inflammation,thereby attenuating the development of biliary strictures (46). We hypothesize that LCBDE injures the CBD and creates necrosis and inflammation at the site of the CBD incision, which in turn leads to the development of biliary strictures. However, this needs to be evaluated with longer follow-up and deeper studies.

Mortality was defined as the rate of death occurring in patients within 30 days after surgery. Meta-analysis showed that the mortality rate was lower in the LTCBDE group compared with the LCBDE group, and the difference between the two groups was statistically significant (*P* = 0.04). In our meta-analysis, only 2 studies were included, a small sample size. However, it is worth noting that all 4 deaths were from the LCBDE group, and 2 were due to postoperative pneumonia and thus death. Relevant studies have shown that postoperative pulmonary complications, especially atelectasis and pneumonia, are the main causes of postoperative morbidity and mortality (47). Therefore, we should pay attention to and evaluate the respiratory function of patients before and after surgery and provide appropriate interventions to avoid the occurrence of postoperative pneumonia.

Analysis of the present meta-data showed that the incidence of stone recurrence was significantly lower in the LTCBDE group than in the LCBDE group, and the difference between the two groups was statistically significant(*P* = 0.007). According to our summarized indications, patients with LCBDE usually have a dilated CBD and have larger stones. In some studies (26, 27), significant risk factors for stone recurrence were bile duct diameter and the presence of periportal diverticula. Bile duct diameter is a significant predictor of bile duct stone recurrence. Some authors consider bile duct diameter ≥15 mm as a risk factor for stone recurrence. In addition, large stones usually require mechanical lithotripsy. Mechanical lithotripsy may increase the risk of recurrence because even some missed small stone fragments may cause stone reaggregation (48). Some studies have found that the T-tube is a foreign body and stone recurrence may be associated with bile pigment and bile salt deposition around the T-tube (49). These may be the reasons for the higher rate of stone recurrence in the LCBDE group than in the LTCBDE group.

### Limitations

The strength of this meta-analysis is that it provides a comprehensive analysis of LTCBDE and LCBDE, comparing the differences in individual postoperative complications between LTCBDE and LCBDE. To the best of our knowledge, this is one of the few meta-analyses exploring these two techniques. Of course, we need to see that this meta-analysis has some limitations. Firstly, we excluded 3 non-English language papers, and publication and selection bias may have been an issue. Secondly some of the outcome indicators such as bile leak, pancreatitis and other complications do not have uniform diagnostic criteria, which may cause errors due to the subjective judgment of doctors. Thirdly, there was significant heterogeneity among studies in terms of operative time and postoperative hospital stay. Although we used a random-effects model to reduce the effect of heterogeneity and performed a sensitivity analysis, it could not be completely eliminated. Fourth, due to the insufficiently large study sample size, this study has not yet been able to analyze and compare the TTD subgroup and PDC subgroup with the LTCBDE group separately. More and larger multicenter randomized controlled trials with longer follow-up are needed to provide reliable data in the future.

## Conclusion

Compared with LCBDE, LTCBDE not only improved the stone removal rate, but also significantly reduced the operative time, postoperative hospital stay, postoperative complications, and lowered the rates of residual stones, bile leak, and recurrent stones, in addition to the fact that there was no difference in any of the postoperative pancreatitis and biliary stricture. LTCBDE is safe, efficient, and of great clinical significance, and is worth promoting to some patients.

## Data Availability

The original contributions presented in the study are included in the article/Supplementary Material, further inquiries can be directed to the corresponding author.

## References

[1] Zhu J Sun G Hong L Li X Li Y Xiao W. Laparoscopic common bile duct exploration in patients with previous upper abdominal surgery. Surg Endosc. (2018) 32(12):4893–9. 10.1007/s00464-018-6248-3 29869082

[2] Liu Q Li T Feng Z Han W. Medium and long-term complications difference between laparoscopic transcystic common bile duct exploration versus endoscopic sphincterotomy against choledocholithiasis. Medicine (Baltimore). (2021) 100(3):e24104. 10.1097/MD.0000000000024104 33546017 PMC7837953

[3] Yin Z Xu K Sun J Zhang J Xiao Z Wang J Is the end of the T-tube drainage era in laparoscopic choledochotomy for common bile duct stones is coming? A systematic review and meta-analysis. Ann Surg. (2013) 257(1):54–66. 10.1097/SLA.0b013e318268314b 23059495

[4] Furukawa T. Subtyping of IPMN. Methods Mol Biol. (2019) 1882:1–8. 10.1007/978-1-4939-8879-2_1 30378039

[5] Farrell RJ Noonan N Mahmud N Morrin MM Kelleher D Keeling PW. Potential impact of magnetic resonance cholangiopancreatography on endoscopic retrograde cholangiopancreatography workload and complication rate in patients referred because of abdominal pain. Endoscopy. (2001) 33(8):668–75. 10.1055/s-2001-16218 11490382

[6] Zhang R Liu J Li H Zeng Q Wu S Tian H. Evaluation of therapeutic efficacy, safety and economy of ERCP and LTCBDE in the treatment of common bile duct stones. Front Physiol. (2022) 13:949452. 10.3389/fphys.2022.949452 36091409 PMC9452837

[7] Zaigham H Enochsson L Ottosson J Regnér S. Laparoscopic transcystic common bile duct exploration versus transgastric endoscopic retrograde cholangiography during cholecystectomy after roux-en-Y gastric bypass. Surg Obes Relat Dis. (2023) 19(8):882–8. 10.1016/j.soard.2023.01.023 36870871

[8] Luo D Wan X Liu J Tong T. Optimally estimating the sample mean from the sample size, median, mid-range, and/or mid-quartile range. Stat Methods Med Res. (2018) 27(6):1785–805. 10.1177/0962280216669183 27683581

[9] Martin IJ Bailey IS Rhodes M O'Rourke N Nathanson L Fielding G. Towards T-tube free laparoscopic bile duct exploration. Ann Surg. (1998) 228(1):29–34. 10.1097/00000658-199807000-00005 9671063 PMC1191424

[10] Rhodes M Sussman L Cohen L Lewis MP. Randomised trial of laparoscopic exploration of common bile duct versus postoperative endoscopic retrograde cholangiography for common bile duct stones. Lancet. (1998) 351(9097):159–61. 10.1016/S0140-6736(97)09175-7 9449869

[11] Cuschieri A Lezoche E Morino M Croce E Lacy A Toouli J E.A.E.S. multicenter prospective randomized trial comparing two-stage vs single-stage management of patients with gallstone disease and ductal calculi. Surg Endosc. (1999) 13(10):952–7. 10.1007/s004649901145 10526025

[12] Lauter DM Froines EJ. Laparoscopic common duct exploration in the management of choledocholithiasis. Am J Surg. (2000) 179(5):372–4. 10.1016/S0002-9610(00)00368-8 10930482

[13] Waage A Strömberg C Leijonmarck CE Arvidsson D. Long-term results from laparoscopic common bile duct exploration. Surg Endosc. (2003) 17(8):1181–5. 10.1007/s00464-002-8937-0 12739114

[14] Paganini AM Guerrieri M Sarnari J De Sanctis A D'Ambrosio G Lezoche G Thirteen years’ experience with laparoscopic transcystic common bile duct exploration for stones. Surg Endosc. (2007) 21(1):34–40. 10.1007/s00464-005-0286-3 17111284

[15] Topal B Aerts R Penninckx F. Laparoscopic common bile duct stone clearance with flexible choledochoscopy. Surg Endosc. (2007) 21(12):2317–2. 10.1007/s00464-007-9577-1 17943379

[16] ElGeidie AA ElShobary MM Naeem YM. Laparoscopic exploration versus intraoperative endoscopic sphincterotomy for common bile duct stones: a prospective randomized trial. Dig Surg. (2012) 28(5-6):424–31. 10.1159/000331470 22236538

[17] Grubnik VV Tkachenko AI Ilyashenko VV Vorotyntseva KO. Laparoscopic common bile duct exploration versus open surgery: comparative prospective randomized trial. Surg Endosc. (2012) 26(8):2165–71. 10.1007/s00464-012-2194-7 22350244

[18] Chen XM Zhang Y Cai HH Sun DL Liu SY Duan YF Transcystic approach with micro-incision of the cystic duct and its confluence part in laparoscopic common bile duct exploration. J Laparoendosc Adv Surg Tech A. (2013) 23(12):977–81. 10.1089/lap.2013.0309 24138388

[19] Poh B Cashin P Bowers K Ackermann T Tay YK Dhir A Management of choledocholithiasis in an emergency cohort undergoing laparoscopic cholecystectomy: a single-centre experience. HPB. (2014) 16(7):629–34. 10.1111/hpb.12187 24246139 PMC4105900

[20] Hongjun H Yong J Baoqiang W. Laparoscopic Common Bile Duct Exploration. Surg Laparosc Endosc Percutan Tech. (2015) 25(3):218–22. 10.1097/SLE.0000000000000133 25799258

[21] Zhang W Xu G Huang Q Luo K Dong Z Li J Treatment of gallbladder stone with common bile duct stones in the laparoscopic era. BMC Surg. (2015) 15:7. 10.1186/1471-2482-15-7 25623774 PMC4417333

[22] Aawsaj Y Light D Horgan L. Laparoscopic common bile duct exploration: 15-year experience in a district general hospital. Surg Endosc. (2016) 30(6):2563–6. 10.1007/s00464-015-4523-0 26307600

[23] Mattila A Mrena J Kellokumpu I. Cost-analysis and effectiveness of one-stage laparoscopic versus two-stage endolaparoscopic management of cholecystocholedocholithiasis: a retrospective cohort study. BMC Surg. (2017) 17(1):79. 10.1186/s12893-017-0274-2 28683735 PMC5501265

[24] Quaresima S Balla A Guerrieri M Campagnacci R Lezoche E Paganini AM. A 23 year experience with laparoscopic common bile duct exploration. HPB. (2017) 19(1):29–35. 10.1016/j.hpb.2016.10.011 27890483

[25] Al-Temimi MH Rangarajan S Chandrasekaran B Kim EG Trujillo CN Mousa AF Predictors of failed transcystic laparoscopic common bile duct exploration: analysis of multicenter integrated health system database. J Laparoendosc Adv Surg Tech A. (2019) 29(3):360–5. 10.1089/lap.2018.0360 30207856

[26] Al-Ardah M Barnett RE Morris S Abdelrahman T Nutt M Boyce T Lessons learnt from the first 200 unselected consecutive cases of laparoscopic exploration of common bile duct stones at a district general hospital. Surg Endosc. (2021) 35(11):6268–77. 10.1007/s00464-020-08127-w 33140155

[27] Guo T Wang L Xie P Zhang Z Huang X Yu Y. Surgical methods of treatment for cholecystolithiasis combined with choledocholithiasis: six years’ experience of a single institution. Surg Endosc. (2022) 36(7):4903–11. 10.1007/s00464-021-08843-x 34731303 PMC9160127

[28] Nassar AHM Ng HJ Katbeh T Cannings E. Conventional surgical management of bile duct stones. Ann Surg. (2022) 276(5):e493–501. 10.1097/SLA.0000000000004680 33351482

[29] Zhu J Han W Zhang Z Guo W. Microincision of the cyst duct is safe and effective for the failed laparoscopic transcystic common bile duct exploration. Indian J Surg. (2022) 84:1263–8. 10.1007/s12262-022-03304-8

[30] Zhu HY Xu M Shen HJ Yang C Li F Li KW A meta-analysis of single-stage versus two-stage management for concomitant gallstones and common bile duct stones. Clin Res Hepatol Gastroenterol. (2015) 39(5):584–93. 10.1016/j.clinre.2015.02.002 25936687

[31] Li T Wen J Bie L Gong B. Comparison of the long-term outcomes of endoscopic papillary large balloon dilation alone versus endoscopic sphincterotomy for removal of bile duct stones. Gastroenterol Res Pract. (2018) 2018:6430701. 10.1155/2018/6430701 30057600 PMC6051268

[32] Vezakis A Fragulidis G Polydorou A. Endoscopic retrograde cholangiopancreatography-related perforations: diagnosis and management. World J Gastrointest Endosc. (2015) 7(14):1135–41. 10.4253/wjge.v7.i14.1135 26468337 PMC4600179

[33] Wang X Wang X Sun H Ren G Wang B Liang S Endoscopic papillary large balloon dilation reduces further recurrence in patients with recurrent common bile duct stones: a randomized controlled trial. Am J Gastroenterol. (2022) 117(5):740–7. 10.14309/ajg.0000000000001690 35191430

[34] Pan L Chen M Ji L Zheng L Yan P Fang J The safety and efficacy of laparoscopic common bile duct exploration combined with cholecystectomy for the management of cholecystocholedocholithiasis. Ann Surg. (2018) 268(2):247–53. 10.1097/SLA.0000000000002731 29533266

[35] Decker G Borie F Millat B Berthou JC Deleuze A Drouard F One hundred laparoscopic choledochotomies with primary closure of the common bile duct. Surg Endosc. (2003) 17(1):12–8. 10.1007/s00464-002-9012-6 12364990

[36] Navaratne L Martinez Isla A. Transductal versus transcystic laparoscopic common bile duct exploration: an institutional review of over four hundred cases. Surg Endosc. (2021) 35(1):437–48. 10.1007/s00464-020-07522-7 32246237

[37] Xia HT Liu Y Jiang H Yang T Liang B Zeng JP A novel laparoscopic transcystic approach using an ultrathin choledochoscope and holmium laser lithotripsy in the management of cholecystocholedocholithiasis: an appraisal of their safety and efficacy. Am J Surg. (2018) 215(4):631–5. 10.1016/j.amjsurg.2017.05.020 28624229

[38] Xia HT Liang B Liu Y Yang T Zeng JP Dong JH. Ultrathin choledochoscope improves outcomes in the treatment of gallstones and suspected choledocholithiasis. Expert Rev Gastroenterol Hepatol. (2016) 10(12):1409–13. 10.1080/17474124.2016.1250623 27796141

[39] Feng Q Huang Y Wang K Yuan R Xiong X Wu L. Laparoscopic transcystic common bile duct exploration: advantages over laparoscopic choledochotomy. PLoS One. (2016) 11(9):e0162885. 10.1371/journal.pone.0162885 27668730 PMC5036868

[40] Pang L Zhang Y Wang Y Kong J. Transcystic versus traditional laparoscopic common bile duct exploration: its advantages and a meta-analysis. Surg Endosc. (2018) 32(11):4363–76. 10.1007/s00464-018-6286-x 29943056

[41] Koch M Garden OJ Padbury R Rahbari NN Adam R Capussotti L Bile leakage after hepatobiliary and pancreatic surgery: a definition and grading of severity by the international study group of liver surgery. Surgery. (2011) 149(5):680–8. 10.1016/j.surg.2010.12.002 21316725

[42] Zhang D Ma Y Sun W Wang N Liu Z Lu Z. Primary suture for patients of bile duct stones after laparoscopic biliary tract exploration: a retrospective cohort study. Updates Surg. (2023) 75(4):897–903. 10.1007/s13304-023-01451-5 36749505

[43] Wills VL Gibson K Karihaloot C Jorgensen JO. Complications of biliary T-tubes after choledochotomy. ANZ J Surg. (2002) 72(3):177–80. 10.1046/j.1445-2197.2002.02308.x 12071447

[44] Wang Q Zhang X Sun L Yang N. Primary two-layered closure of the common bile duct reduces postoperative bile leakage after laparoscopic common bile duct exploration. J Laparoendosc Adv Surg Tech A. (2021) 31(11):1274–8. 10.1089/lap.2020.0768 33347783

[45] Tenner S Baillie J DeWitt J Vege SS. American college of gastroenterology guideline: management of acute pancreatitis. Am J Gastroenterol. (2013) 108(9):1400–15. 10.1038/ajg.2013.218 23896955

[46] Brooks-Brunn JA. Postoperative atelectasis and pneumonia. Heart Lung. (1995) 24(2):94–115. 10.1016/S0147-9563(05)80004-4 7759282

[47] Maatman TK Ceppa EP Fogel EL Easier JJ Gromski MA House MG Biliary stricture after necrotizing pancreatitis. Ann Surg. (2022) 276(1):167–72. 10.1097/SLA.0000000000004470 33086318 PMC10157776

[48] Sugiyama M Atomi Y. Risk factors predictive of late complications after endoscopic sphincterotomy for bile duct stones: long-term (more than 10 years) follow-up study. Am J Gastroenterol. (2002) 97(11):2763–7. 10.1111/j.1572-0241.2002.07019.x 12425545

[49] Rienhoff WF. Primary closure of the common duct. Ann Surg. (1960) 151(2):255–60. 10.1097/00000658-196002000-00016 17859621 PMC1613288

