# Supplementary material for: Comparison of the efficacy of LTCBDE and LCBDE for common bile duct stones: a systematic review and meta-analysis
Source: Front Surg. 2025 Jan 8;11:1412334. doi: 10.3389/fsurg.2024.1412334 (PMC11750767; doi:10.3389/fsurg.2024.1412334)
Supplement: Supplementary file 1 [file Supplementaryfile1.docx]

**Table S1** Clinical outcomes of the included studies

| Study | CBDs clearance | |  | Operative time (Mean±SD,min) | |  | Postoperative hospital stay (Mean±SD,day) | |
| --- | --- | --- | --- | --- | --- | --- | --- | --- |
|  | LTCBDE | LCBDE |  | LTCBDE | LCBDE |  | LTCBDE | LCBDE |
|  |  | (TTD) / (PDC) |  |  | TTD PDC |  |  | TTD PDC |
| Martin 1998 | 154/158 | (58/61)/(53/55) |  | 84.74±38.59^a^ | 136.58±54.99^a^/128.72±44.96^a^ |  | 2.4±2.2^a^ | 6.2±7.3^a^/2.4±1.5^a^ |
| Rhodes 1998 | 23/28 | 7/12 |  | - | - |  | - | - |
| Cuschieri 1999 | 45/56 | (45/53)/ - |  | - | - |  | - | - |
| Lauter 2000 | 26/26 | - /(21/25) |  | 168.15±64.37^b^ | -/276.74±99.53^b^ |  | 2.5±1.5^b^ | -/2.9±1.78^b^ |
| Waage 2003 | 104/110 | (50/52)/ - |  | 213.43±99.13^a^ | 213.43±99.13^a^/- |  | 2.8±3.6^b^ | 6.2±3.1^b^/- |
| Paganini 2007 | 185/191 | 126/138 |  | - | - |  | - | - |
| Topal 2007 | 77/83 | 28/30 |  | 82.62±43.17^a^ | 82.62±43.17^a^/ |  | 3.3±4.9^a^ | 8.9±7.84^a^ |
| ElGeidie 2011 | 56/57 | 47/49 |  | - | - |  | - | - |
| Grubnik 2012 | 72/76 | 58/62 |  | 62±16.67^b^ | 62±16.67^b^ |  | 3.4 ± 1.7 | 7.6 ± 2.5 |
| Chen 2013 | - | - |  | 100±30.4 | 100±30.4 |  | 3.6±0.9 | 7.9±1.0 |
| Poh 2014 | 44/80 | 3/3 |  | - | - |  | 5.7±4.5^c^ | 6.17±2.5^c^ |
| Huang 2015 | - | - |  | 91.9±34.21 | -/96.13± 32.25 |  | - | - |
| Zhang 2015 | 228/237 | (45/46)/(44/47) |  | 76.0±20.2 | 126.4±29.5/106.0±22.6 |  | 3.9±1.8 | 8.4±2.8/5.1±1.6 |
| Aawsaj 2016 | - | - |  | 96±39.04^b^ | 137±30.17^b^ |  | 2±1.5^b^ | 6±4.31^b^ |
| Mattila 2017 | 59/64 | 27/33 |  | 140±106.71^c^ | 165±168.55^c^ |  | 2±1.52^c^ | 5±4.7^c^ |
| Quaresima 2017 | 208/214 | 156/170 |  | 127±69 | 191 ± 74 |  | 5±1.6^a^ | 7.2±2.6^a^ |
| Al-Temimi 2019 | 91/103 | 11/12 |  | - | - |  | 3.6±2.5 | 5.0±3.2 |
| Al-Ardah 2021 | 109/111 | 66/68 |  | 162±48 | 234±70 |  | 3.5±3.3 | 9.4±12.1 |
| Guo 2022 | 278/280 | - /(473/479) |  | 92.31±10.26 | -/99.09±8.46 |  | 6.92±1.71 | -/0.74±5.30 |
| Nassar 2022 | 857/870 | 429/448 |  | 117.19±37.65^a^ | 143.19±96.15^a^ |  | - | - |
| Zhu 2022 | 66/68 | 63/68 |  | 126.30±16.53^c^ | 151.08±15.90^c^ |  | 3.71±3.03^c^ | 5±1.51^c^ |

CBDs: Common bile duct stones; a: transformed from the median (range). b: transformed from the average (range). c: transformed from median (IQR).
