# Supplementary material for: Comparison of the efficacy of LTCBDE and LCBDE for common bile duct stones: a systematic review and meta-analysis
Source: Front Surg. 2025 Jan 8;11:1412334. doi: 10.3389/fsurg.2024.1412334 (PMC11750767; doi:10.3389/fsurg.2024.1412334)
Supplement: Supplementary file 2 [file Supplementaryfile2.docx]

**Table S2** Complications of the included studies

| Study | Residual stone | |  | Bile leakage | |  | Pancreatitis | |  | Mortality | |
| --- | --- | --- | --- | --- | --- | --- | --- | --- | --- | --- | --- |
|  | LTCBDE | LCBDE |  | LTCBDE | LCBDE |  | LTCBDE | LCBDE |  | LTCBDE | LCBDE |
|  |  | TTD / PDC |  |  | TTD / PDC |  |  | TTD / PDC |  |  | TTD / PDC |
| Martin 1998 | 4 | 3/2 |  | 1 | 3/3 |  | 1 | 0/0 |  | - | - |
| Rhodes 1998 | - | - |  | - | - |  | - | - |  | - | - |
| Cuschieri 1999 | - | - |  | - | - |  | - | - |  | - | - |
| Lauter 2000 | 0 | -/4 |  | - | - |  | - | - |  | - | - |
| Waage 2003 | 6 | 2/- |  | 1 | 0/- |  | - | - |  | - | - |
| Paganini 2007 | 6 | 12/- |  | 2 | 0/- |  | 1 | 0/- |  | - | - |
| Topal 2007 | 6 | 2 |  | - | - |  | - | - |  | - | - |
| ElGeidie 2011 | 1 | 2 |  | 2 | 3 |  | - | - |  | - | - |
| Grubnik 2012 | 4 | 4 |  | - | - |  | - | - |  | - | - |
| Chen 2013 | - | - |  | 1 | 10 |  | - | - |  | - | - |
| Poh 2014 | 36 | 0 |  | - | - |  | - | - |  | - | - |
| Huang 2015 | - | - |  | 1 | -/5 |  | - | - |  | - | - |
| Zhang 2015 | 9 | 1/3 |  | 3 | 5/2 |  | 1 | 0/1 |  | - | - |
| Aawsaj 2016 | - | - |  | 0 | 14 |  | - | - |  | - | - |
| Mattila 2017 | 5 | 6 |  | 2 | 3 |  | 1 | 0 |  | 0 | 1 |
| Quaresima 2017 | 6 | 14 |  | 3 | 3 |  | 1 | 2 |  | - | - |
| Al-Temimi 2019 | 12 | 1 |  | - | - |  | 2 | 0 |  | - | - |
| Al-Ardah 2021 | 2 | 2 |  | 2 | 3 |  | - | - |  | - | - |
| Guo 2022 | 2 | -/6 |  | 3 | -/5 |  | 0 | -/1 |  | - | - |
| Nassar 2022 | 9 | 19 |  | 13 | 18 |  | 13 | 5 |  | 0 | 3 |
| Zhu 2022 | 2 | 5 |  | 2 | 2 |  | 1 | 1 |  | - | - |

| Study | Residual stone | |  | Bile leakage | |  | Pancreatitis | |  | Mortality | |
| --- | --- | --- | --- | --- | --- | --- | --- | --- | --- | --- | --- |
|  | LTCBDE | LCBDE |  | LTCBDE | LCBDE |  | LTCBDE | LCBDE |  | LTCBDE | LCBDE |
|  |  | TTD / PDC |  |  | TTD / PDC |  |  | TTD / PDC |  |  | TTD / PDC |
| Martin 1998 | 4 | 3/2 |  | 1 | 3/3 |  | 1 | 0/0 |  | - | - |
| Rhodes 1998 | - | - |  | - | - |  | - | - |  | - | - |
| Cuschieri 1999 | - | - |  | - | - |  | - | - |  | - | - |
| Lauter 2000 | 0 | -/4 |  | - | - |  | - | - |  | - | - |
| Waage 2003 | 6 | 2/- |  | 1 | 0/- |  | - | - |  | - | - |
| Paganini 2007 | 6 | 12/- |  | 2 | 0/- |  | 1 | 0/- |  | - | - |
| Topal 2007 | 6 | 2 |  | - | - |  | - | - |  | - | - |
| ElGeidie 2011 | 1 | 2 |  | 2 | 3 |  | - | - |  | - | - |
| Grubnik 2012 | 4 | 4 |  | - | - |  | - | - |  | - | - |
| Chen 2013 | - | - |  | 1 | 10 |  | - | - |  | - | - |
| Poh 2014 | 36 | 0 |  | - | - |  | - | - |  | - | - |
| Huang 2015 | - | - |  | 1 | -/5 |  | - | - |  | - | - |
| Zhang 2015 | 9 | 1/3 |  | 3 | 5/2 |  | 1 | 0/1 |  | - | - |
| Aawsaj 2016 | - | - |  | 0 | 14 |  | - | - |  | - | - |
| Mattila 2017 | 5 | 6 |  | 2 | 3 |  | 1 | 0 |  | 0 | 1 |
| Quaresima 2017 | 6 | 14 |  | 3 | 3 |  | 1 | 2 |  | - | - |
| Al-Temimi 2019 | 12 | 1 |  | - | - |  | 2 | 0 |  | - | - |
| Al-Ardah 2021 | 2 | 2 |  | 2 | 3 |  | - | - |  | - | - |
| Guo 2022 | 2 | -/6 |  | 3 | -/5 |  | 0 | -/1 |  | - | - |
| Nassar 2022 | 9 | 19 |  | 13 | 18 |  | 13 | 5 |  | 0 | 3 |
| Zhu 2022 | 2 | 5 |  | 2 | 2 |  | 1 | 1 |  | - | - |
