# Supplementary material for: Comparison of the efficacy of LTCBDE and LCBDE for common bile duct stones: a systematic review and meta-analysis
Source: Front Surg. 2025 Jan 8;11:1412334. doi: 10.3389/fsurg.2024.1412334 (PMC11750767; doi:10.3389/fsurg.2024.1412334)
Supplement: Supplementary file 3 [file Supplementaryfile3.docx]

**Table S3** Technical processes and follow-up of the 21 included studies

| Study | Technical approaches | |  | Indication | |  | Follow-up |
| --- | --- | --- | --- | --- | --- | --- | --- |
|  | LTCBDE | LCBDE |  | LTCBDE | LCBDE |  |  |
| Martin 1998 | A helical 4.5-mm ureteric stone basket | 4-0PDS sutures;  Antegrade stents |  | Small stones (<1 cm) distal to the cystic duct-common duct junction | Large stones (>1 cm) ; stones that remained above the cystic duct-common duct junction; transcystic exploration failed |  | **－** |
| Rhodes 1998 | Reddick-Olsen cholangiogram forceps | Closed after a pig-tail stent; T-tube inserted |  | Less than 9 mm in diameter in the  common bile duct | Larger stones, or stones in the common hepatic duct; transcysticexploration failed |  | **－** |
| Cuschieri 1999 | Small nonoccluding  Stones | Large or occluding stones |  | **－** | **－** |  | **－** |
| Lauter 2000 | Through the cystic ductotomy with removal of stones either via the cystic duct; by fragmentation and placement into the duodenum | Via choledochotomy  involved stone  removal through an incision in the common duct followed |  | **－** | **－** |  | The mean follow-up time was 27 months (range, 2-62 months) |
| Waage 2003 | A balloon catheter with a diameter of 8 mm and a balloon length of 6 cm | Longitudinal incision; absorbable monofilament interrupted sutures. |  | **－** | Stones larger than 8 mm or proximal to the CBD;unfavourable anatomy of the cystic duct–CBD junction was identified |  | From 6 to 72 months (median, 36 months) |
| Paganini 2007 | **－** | Blind basketing; a complete choledochoscopy is performed with a 7.5 Fr; choice to position an external biliary drain |  | **－** | CBD stones larger than the lumen of cystic duct; >5 CBD stones; low and medial cystic duct-CBD junction; common hepatic duct stones |  | Median: 118.0 months; range: 17.6–168 months |

| Study | Technical approaches | |  | Indication | |  | Follow-up |
| --- | --- | --- | --- | --- | --- | --- | --- |
|  | LTCBDE | LCBDE |  | LTCBDE | LCBDE |  |  |
| Topal  2007 | Dissect and clip the cystic duct; FCD through a small incision in  the cystic duct | Stones could not be extracted through the cystic duct |  | **－** | **－** |  | **－** |
| ElGeidie 2011 | Intraoperative cholangio-graphy (IOC); transcystic CBD  Exploration | Intraoperative cholangiography (IOC); choledochotomy |  | **－** | CBD>10 mm,stones>10 mm in size or multiple stones>4, in cases of proximal location, unfavorable cystic duct-CBD junction |  | **－** |
| Grubnik 2012 | Intraoperative cholangiography;  3 mm flexible choledochoscope;  a complete cholangiography | Exposure of the porta hepatis; cut anterior wall about10–20 mm;  choledochoscope control; absorbable running suture |  | **－** | Impacted stones; stones  diameter >5–7 mm; multiplestones;  unfavorable anatomy |  | **－** |
| Chen  2013 | The confluence part was cut open 3–5 mm at the supra and inferior margins | A longitudinal incision; T tube of appropriate size |  | **－** | Cystic duct anatomy, presence of stones of diameter>0.6 cm; a  large number of stones |  | All patients were routinely assessed 12 months after discharge |
| Poh  2014 | A longitudinal incision | **－** |  | **－** | **－** |  | The median follow-up time was 17 months |
| Huang 2015 | A longitudinal incision; choledochoscope; washed via suction with a soft pipe; ligated with a Hem-o-lock | **－** |  | **－** | **－** |  | Routinely assessed for 12 months after hospital discharge |
| Zhang  2015 | Dilatation was carried out first  with blunt | Dissect and clip the cystic duct; Further dissection towards CBD |  | Stones<9 mm,<5 in number, and cystic duct lateral entrance to CBD | **－** |  | Using ultrasound was carried out in 3 to 24 months after discharge |

| Study | Technical approaches | |  | Indication | |  | Follow-up |
| --- | --- | --- | --- | --- | --- | --- | --- |
|  | LTCBDE | LCBDE |  | LTCBDE | LCBDE |  |  |
| Aawsaj 2016 | **－** | A longitudinal incision using a Berci knife and micro-scissors; closed primarily or over a T tube |  | **－** | CBD in the diameter of ≥9 mm;stone size ≥9 mm;stone number>5, failure of LTSE, and proximal bile ductal  calculi |  | Mean 6 months(range 3–36 months) |
| Mattila 2017 | Dormia basket, Fogarty balloon catheter or flushing and pushing techniques | **－** |  | Transcystic CBD exploration was preferred for stones less than 7–8 mm in diameter; the cystic junction was lateral | Stones>7–8 mm; multiple stones;CBD stones were situated above the cystic duct junction; the cystic junction was posterior, or TC approach failed |  | **－** |
| Quaresima 2017 | **－** | The CBD was opened transversely |  | Adilated cystic duct, joining the CBD on its lateral side; <4 ducta stonesl; ductal stones <5 mm in size and smaller than the size of the cystic duct, located only in the CBD and not in the common hepatic duct | CBD diameter was atleast 10 mm. CBDstones larger than the size of the cystic duct; >4 ductal stones; low and medial cystic duct – CBD junction, stones located in the common hepatic duct |  | Mean189±105 months |
| Al-Temimi  2019 | A 4F Fogarty balloon was inserted; over-the-wire dilator or over-the-wire balloon dilator | **－** |  | **－** | **－** |  | The mean time was13.8 (0.5-48.5) months |
| Al-Ardah 2021 | Introduced through a transverse cystic ductotomy for the transcystic approach and via a longitudinal supra-duodenal CBD choledochotomy | 4–0 Vicryl suture either interrupted or continuous in accordance with the operating surgeon preference |  | **－** | **－** |  | The mean time was 48±42 months |

| Study | Technical approaches | |  | Indication | |  | **Follow-up** |
| --- | --- | --- | --- | --- | --- | --- | --- |
|  | LTCBDE | LCBDE |  | LTCBDE | LCBDE |  |  |
| Guo 2022 | The cystic duct was cut transversely at a distance of 1–2 cm to CBD, after which a catheter or balloon was used to dilate the cystic duct; T-shaped incision at the confuence of the cystic duct and CBD and used electrohydraulic lithotripsy or biopsy forceps for stone fragmentation | Choledochotomy performed, the length is 1–1.5 cm,ENBD tube was slowly and gently sent to 1–2 cm above the choledochal incision; closed with a 4–0 absorbable suture |  | Cystic duct diameter  > 3 mm, number of common bile duct stones <5 or stone diameter < Patients with 2 cm | **－** |  | 6 to 72 months |
| Nassar 2022 | **－** | The choledochotomy is closed with small bites of interrupted 3-0 Vicryl to reduce the risk of stricturing. . A size 6Fr tube is inserted and the CD ligated around it before closing the choledochotomy. |  | **－** | **－** |  | 1-24 years |
| Zhu  2022 | A transverse incision was made in the lateral wall of the cystic duct after the bile duct had been clipped 1 cm upstream of the CBD,longitudinal incision of the cystic duct at the confuence with CBD with the extension of about 3–5 mm at the lateral margin of the CBD;closed with interrupted 4-0 Vicryl | **－** |  | **－** | **－** |  | Median follow-up of 7.4 years |
