# Supplementary material for: Comparison of the efficacy of LTCBDE and LCBDE for common bile duct stones: a systematic review and meta-analysis
Source: Front Surg. 2025 Jan 8;11:1412334. doi: 10.3389/fsurg.2024.1412334 (PMC11750767; doi:10.3389/fsurg.2024.1412334)
Supplement: Supplementary file 4 [file Supplementaryfile4.docx]

**Table S4** Assessment of study quality based on the modified Newcastle–Ottawa scale

| Study | Is the Case Definition Adequate? | Representativeness of the Cases | Selection of Controls | Definition of Controls | Ascertainment of Exposure | Comparability | Assessment of Outcome | Follow-up long enough | Adequacy of Follow Up of Cohorts | Total score |
| --- | --- | --- | --- | --- | --- | --- | --- | --- | --- | --- |
| Martin 1998 | ☆ | ☆ | ☆ |  | ☆ |  | ☆ |  |  | 5 |
| Lauter 2000 | ☆ | ☆ | ☆ | ☆ | ☆ |  | ☆ | ☆ | ☆ | 8 |
| Waage 2003 | ☆ | ☆ | ☆ |  | ☆ |  | ☆ | ☆ |  | 6 |
| Paganini 2007 | ☆ | ☆ |  | ☆ | ☆ |  | ☆ | ☆ |  | 6 |
| Topal 2007 | ☆ | ☆ | ☆ |  | ☆ |  | ☆ |  |  | 5 |
| Chen 2013 | ☆ | ☆ | ☆ | ☆ | ☆ |  | ☆ | ☆ |  | 7 |
| Poh 2014 | ☆ | ☆ | ☆ | ☆ | ☆ |  | ☆ | ☆ | ☆ | 8 |
| Huang 2015 | ☆ | ☆ | ☆ | ☆ | ☆ |  | ☆ | ☆ |  | 7 |
| Zhang 2015 | ☆ | ☆ | ☆ |  | ☆ |  | ☆ | ☆ |  | 6 |
| Aawsaj 2016 | ☆ | ☆ | ☆ |  | ☆ |  | ☆ | ☆ |  | 6 |
| Mattila 2017 | ☆ | ☆ | ☆ | ☆ | ☆ |  | ☆ |  |  | 6 |
| Quaresima 2017 | ☆ | ☆ | ☆ | ☆ | ☆ | ☆ | ☆ | ☆ | ☆ | 9 |
| Al-Temimi 2019 | ☆ | ☆ | ☆ | ☆ | ☆ |  | ☆ | ☆ |  | 7 |
| Al-Ardah 2021 | ☆ | ☆ | ☆ | ☆ | ☆ | ☆ | ☆ | ☆ | ☆ | 9 |
| Guo 2022 | ☆ | ☆ | ☆ | ☆ | ☆ | ☆ | ☆ | ☆ | ☆ | 9 |
| Nassar 2022 | ☆ | ☆ | ☆ | ☆ | ☆ |  | ☆ | ☆ |  | 7 |
| Zhu 2022 | ☆ | ☆ | ☆ | ☆ | ☆ | ☆ | ☆ | ☆ | ☆ | 9 |
