# Supplementary figures and images for: Comparison of the efficacy of LTCBDE and LCBDE for common bile duct stones: a systematic review and meta-analysis
Source: Front Surg. 2025 Jan 8;11:1412334. doi: 10.3389/fsurg.2024.1412334 (PMC11750767; doi:10.3389/fsurg.2024.1412334)

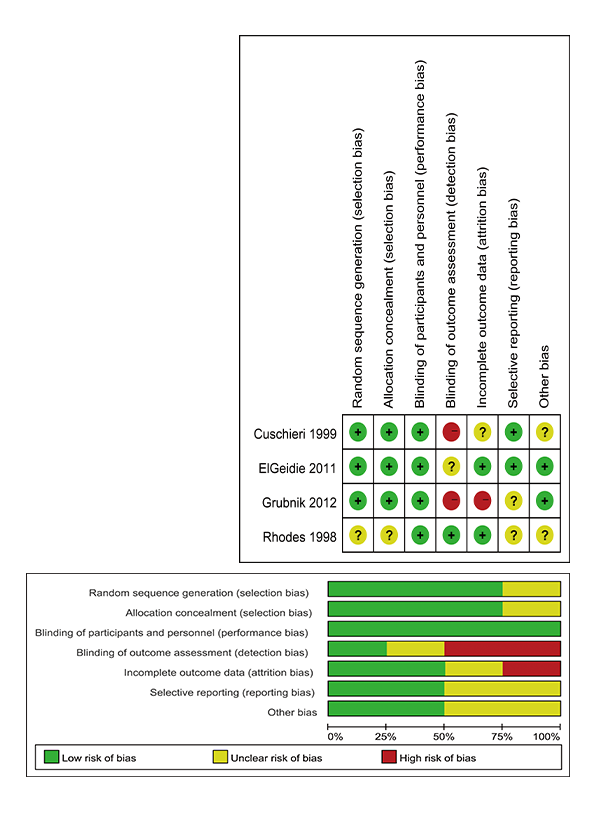

Supplement: Supplementary file 5 [file Image1.tif]

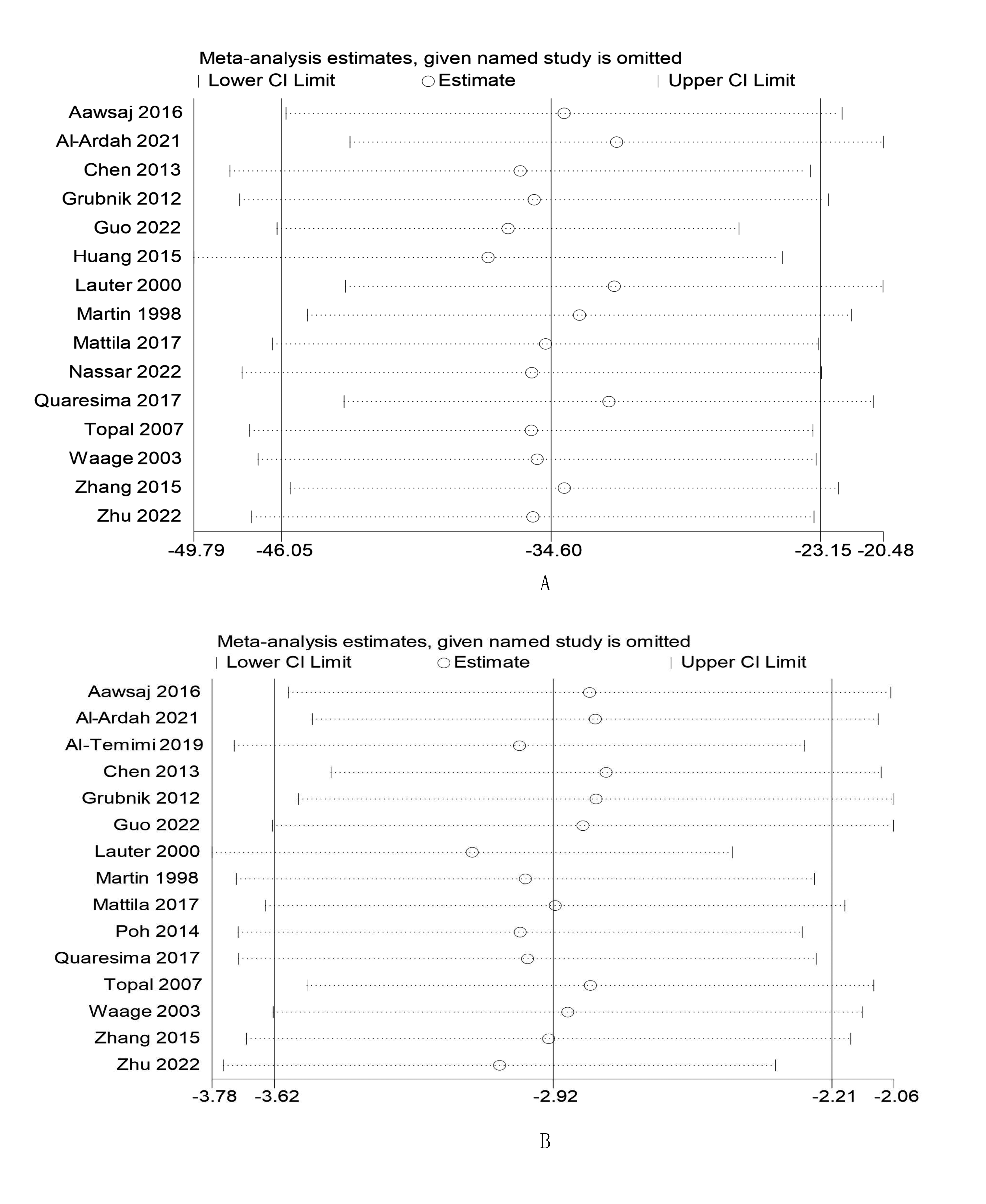

Supplement: Supplementary file 7 [file Image3.tif]
